# Gratuitous Dental Service in the French Primary School

**Published:** 1881-12

**Authors:** 


Gratuitous Dental Service in the French Primary Schools.—We have received a letter from M. E. Taillebois, dentist, of Paris, enclosing a copy of a petition to the Municipal Council of that city, advocating the establishment of a gratuitous dental service in the French primary schools.

The importance of such a sanitary work can hardly be over-estimated. Everyone knows the uniformity with which everything relating to the preservation and health of the teeth is neglected by the poorer classes. It is in order to avoid the bad results of this neglect that Mr. Taillebois proposes to create his free dental service.

It is proposed that the dentists appointed to this duty shall give their services without compensation, and as an evidence of his disinterestedness, the author of the project offers to establish and conduct the service in the first and second arrondissements of Paris at his own expense.

